# Pathophysiological role of zinc in ischemic brain injury

**DOI:** 10.18632/oncotarget.14003

**Published:** 2016-12-16

**Authors:** Zhifeng Qi, Ke Jian Liu

**Affiliations:** ^1^ Beijing Geriatric Medical Research Center, Xuanwu Hospital of Capital Medical University, Beijing, China; Department of Pharmaceutical Sciences, University of New Mexico, Albuquerque, New Mexico, USA

**Keywords:** zinc, blood-brain barrier disruption, cerebral ischemia, Neuroscience

Zinc is the second most abundant metal in human body, and a relatively large amount of zinc is found in the brain, indicating its essential role in central nerve system. Zn^2+^ is stored in synaptic vesicles of glutamatergic neurons and is released from the terminals for synaptic signaling. Zinc is also found in zinc containing proteins. It is estimated that the human proteome contains about 3000 zinc-containing proteins serving signaling, catalytic, and structural roles. In zinc-finger proteins, for example, a zinc ion is complexed in a zinc finger motif through four invariant cysteine and/or histidine residues to form a stable structure and conformation, which regulates protein-DNA, protein-RNA, and protein-protein interactions [1].

Twenty years ago, abnormal zinc accumulation was first observed in ischemic neurons, which leads to neuronal injuries after cerebral ischemia [2]. We recently reported that intracellular zinc dramatically elevated in neurons in the first a few hours following cerebral ischemia, resulting in neuronal apoptotic death in a rat stroke model [3]. We also found that zinc overload contributed to mitochondrial dysfunction in ischemic neurons [4]. Removing zinc with a specific zinc chelator (TPEN) reduced zinc accumulation in ischemic neurons and rescued them from cell death [3, 4]. These findings implicate zinc as a potential target to block the cascade of events leading to ischemic injury.

Zinc release from ischemic neurons to extracellular matrix could produce a surrounding environment with a high concentration of zinc after cerebral ischemia. Strong activities of zinc-containing presynaptic terminals may transiently increase local synaptic zinc concentrations up to 300μM, making it available for entry or uptake by neighboring cells [5]. Microdialysis studies confirmed the increase of extracellular zinc in cerebral ischemia models. Our unpublished data showed that reducing zinc in extracellular matrix with a membrane-impermeable zinc chelator, CaEDTA, could reduce ischemia-induced microvessel injury. These findings suggest that extracellular zinc may be a critical mediator of ischemic brain insult.

Astrocytes can take up the excessive extracellular zinc from synaptic cleft or extracellular matrix to maintain zinc homeostasis. However, zinc overload in astrocytes may induce astrocytic cell death. Our study indicated that zinc overload under hypoxic condition caused a dramatic increase in astrocytic cell death in a zinc-concentration-dependent manner [6]. Very interestingly, hypoxia/ reoxygenation markedly decreased zinc transporter ZnT-1 expression to reduce zinc efflux. Together, these results suggest that hypoxia/reoxygenation may impede the zinc efflux from cells, providing a novel mechanism for intracellular free zinc accumulation after ischemic stroke.

To date, almost all research of zinc in brain has focused on its effects on neuronal functions. Little is known about the role of zinc in blood-brain barrier (BBB) disruption after cerebral ischemia. BBB is mainly composed of endothelium of brain microvessels, which are associated with astrocytes, pericytes, neurons, and extracellular matrix. At the early stage of ischemic stroke, high level of BBB permeability is one of the main causes for hemorrhage transformation, preventing wide use of thrombolytic therapy in most ischemic stroke patients.

The interaction between different types of cells plays very important roles in brain physiology and pathophysiology. Neurons are considered as the first responding cells to ischemic stress, because they are very sensitive and vulnerable to the decrease of oxygen and nutrition supply. Therefore, we hypothesized that, as an impaired signal from neurons, zinc release may contribute to BBB disruption during acute ischemic stroke. Our experiments revealed a strong zinc accumulation in isolated microvessels and *in situ* microvessels in the brain of ischemic rats. Treating the animals with a specific zinc chelator (TPEN) at 60-min post ischemia onset remarkably attenuated microvessel permeability in the ischemic rats [7]. This was the first report showing the contribution of zinc to BBB damage after cerebral ischemia, establishing that excessive zinc release and accumulation are critically involved in ischemia-induced neuronal and vascular injury.

Matrix metalloproteinases (MMPs) family is a large family of zinc-containing endopeptidases that play an important role in mediating gelatin degradation and vasculature disruption. Gelatinases MMP-9 and MMP- 2 specifically degrade the tight junction proteins (TJPs), which seal the gap between endothelial cells to keep BBB integrity. Our study further demonstrated that zinc accumulation in microvessels activated the superoxide/ MMP-9/-2 pathway, leading to the loss of tight junction proteins from microvessels and apoptosis of endothelial cells [7]. These findings indicate that the ROS-MMPs pathway contributed to zinc accumulation-induced BBB disruption during ischemia. One possible source of ROS may be NADPH oxidase pathway in cytosol, based on our previous study that NADPH oxidase-derived ROS generation activated MMP-9, leading to BBB injury after cerebral ischemia. Further investigations are warranted to elucidate how zinc accumulates in microvessels following ischemia and the subsequent molecule events leading to BBB disruption.

In summary, research from our lab and others show that cerebral ischemia triggers zinc accumulation in the microvessels, which critically contributes to ischemia-induced BBB disruption via activating ROS-MMPs pathway. Emerging evidence suggests that zinc is a novel target for reducing both neuronal and vascular damage during ischemic stroke.
